# Multicountry cholera outbreak alert in Kenya: Current efforts and recommendations

**DOI:** 10.1097/JS9.0000000000000130

**Published:** 2023-03-24

**Authors:** Heeba Anis, Olivier Uwishema, Ali E. Hamitoglu, Dina Essayli, Sarah El Kassem, Martin S. Rogose, Zeina Al Maaz, Abubakar Nazir

**Affiliations:** a Oli Health Magazine Organization, Research, and Education, Kigali, Rwanda; b Deccan College of Medical Sciences, Hyderabad, Telangana, India; c Clinton Global Initiative University, New York, New York, USA; d Faculty of Medicine, Karadeniz Technical University, Trabzon; e Namik Kemal University Faculty of Medicine, Tekirdağ, Turkey; f Lebanese University Faculty of Medicine; g Faculty of Medicine, Beirut Arab University, Beirut, Lebanon; h Jewish Pathology Laboratory, Nairobi, Kenya; i Department of Medicine, Kind Edward Medical University, Lahore, Pakistan

*Dear Editor*,

Cholera, caused by the secretion of an enterotoxin by the infectious bacteria *Vibrio cholerae* serogroups O1 or O139, is an acute, diarrheal sickness. Each year, 21 000–143 000 people worldwide die from cholera, which affects an estimated 1.3–4 million persons. Most often the infection is asymptomatic or mild, however, untreated cases can result in death within a few hours after severe dehydration^1^.

In many African countries, cholera is still considered an important source of sickness and death^2^. Particularly in Kenya, an escalating outbreak was reported in May 2022, and by the month’s end, 319 cases and two deaths were reported in three counties: Nairobi, Kisumu, and Kiambu^3^. In addition, in October 2022, the Ministry of Health initiated an alert after confirming 61 cholera cases among six counties^4^.

This study aims to emphasize the return of cholera to Kenya and review the disease and relevant recommendations.

## Epidemiology

Cholera is one of the deadliest tropical diseases caused by *Vibrio cholera* and is spread directly from person to person or indirectly from the environment causing approximately three to five million cases worldwide^5^. African countries have been one of the most affected in terms of pandemic cases as over four million cholera cases and 143 000 deaths were communicated to the WHO in the last five decades^5^. An estimated global burden of this disease suggests that 95 000 deaths and approximately two to nine million cases occur annually in cholera-endemic areas^6^. The majority of deaths and cases were reported in Africa as surveillance in several African sites from 2011 to 2013 found an overall incidence of 0.03 cases per 1000 which increased to two cases per 100 in terms of pandemic^6^. Furthermore, across six sub-Saharan African countries from 2010 to 2013 case fatality ratios (CFRs) varied broadly from 0 to 10% with a median of 1%^6^. Kenya is one of the cholera-endemic zones in Africa, this disease is a threat to the public health in the country with multiple cholera outbreaks recorded since 1971^7^. A 14-year (1997–2010) surveillance report stated that suspected clinical cases were 68 522 and 2641 deaths in that period with a CFR of 3.9%^7^. Considering underreporting and weak surveillance systems the actual number of cholera cases in Africa is likely to be much higher than it is officially reported^8^. A trend of cholera cases is being observed in Kenya nowadays too as 319 suspected cases were estimated, including two confirmed cases and two deaths with an estimated CFR of 0.6% as of 12 May 2022^9^. African countries continue to encounter a high number of cholera outbreaks resulting in a high burden of disease and death^10^. Seventeen countries in Africa accounted for 71 000 cases in 2016; constituting 54% of global cases and 1760 deaths constituting 42% of deaths worldwide^10^. Moreover, the average fatality rate in African countries of 2.5% is significantly higher than the intercontinental average of 1.8%^11^.

## Return of cholera to Kenya

The first incidence of cholera to be reported was in 1971. This was followed by a sequel of similar outbreaks in the years 1997–1999, 2007–2010, 2015–2020, and more recently in 2022, respectively^12^.

Since December 2014, Kenya has been experiencing large cholera outbreaks with cyclical epidemics quinquennial. In 2017, Tana River County became the first county to report a cholera outbreak, followed by Garissa County on 2 April 2017 and then later on in nine other counties. The outbreak was reported on 10 October 2016 and was controlled by April 2017. National Public Health Laboratory in Nairobi cultured 25 samples and 18 turned out to be positive for *V. cholerae* Ogawa. A total of 124 cases tested positive for *V. cholera*e in the reference laboratory in the week ending 25 June 2017. A total of 17 597 cumulative data was collected and confirmed between 2015 and 2016 (10 568 cases reported in 2015 and 6448 in 2016)^13^.

On 19 October 2022, Acting Director General for Health Dr Patrick Amoth issued an alert on the cholera outbreak in six counties whose origin was traced to a wedding festival in Kiambu County. The Ministry of Health confirmed 61 cases across the six counties which were distributed as follows: Kiambu (31), Nairobi (17), Murang’a (1), Kajiado (2), Nakuru (2), and Uasin Gishu (8). Of the 61 cases reported, 13 people were hospitalized, while eight were discharged with 40 treated as outpatients. In the recent outbreak, the *Vibrio cholera* O1 Ogawa serotype was isolated by the National Public Health Microbiology Laboratory^2^.

## Diagnosis and management

Cholera is diagnosed by stool specimens through culture and microscopy, PCR, and rapid diagnostic test (RDT)^14^. The first method depends on dark-field staining, where *V. cholerae* size ranges from 1 to 3 μm, and its diameter ranges from 0.5 to 0.8 μm (https://emedicine.medscape.com/article/962643-overview#a2). Moreover, *V. cholerae* produces large smooth circular colonies on thiosulfate-citrate-bile sucrose-agar^5^. Unfortunately, low-resource areas including Kenya lack sophisticated materials for culturing for definitive diagnosis, thus low-cost RDT replaces culturing and detects *V. cholerae* pathogenic serogroups: O1 and O139^15^,^16^. However, targeting serogroup O1 alone is more specific and sensitive than detecting both serogroups^17^. Moreover, PCR shows greater sensitivity than RDT, thus reserving half of the tip extracts from RDT samples for PCR confirmation eliminates the cost burden during sample collection^18^.

WHO and Mayo Clinic suggest rehydration and educating children, pregnant women, and people with immunodeficiencies, that represent high-risk groups^5^ (https://www.mayoclinic.org/diseases-conditions/cholera/diagnosis-treatment/drc-20355293#:~:text=Although%20signs%20and%20symptoms%20of,quickly%20confirm%20a%20cholera%20diagnosis). It is recommended to achieve water sanitation; however, potable water access is a long-term goal in Kenya, so oral vaccines reduce cholera transmission, and the two feasible vaccines are Dukoral and Shanchol, given through two doses^5^. *V. cholerae* is susceptible to first-line antibiotics, but monitoring the usage of drugs helps avoid antimicrobial drug resistance^19^.

## Recommendations

Cholera incidences can be reduced in both high-risk groups as well as in travelers by applying proper sanitation in addition to performing adequate hygiene practices that include: ensuring that the water being used is safe to be consumed by either drinking water from sealed bottles or in cases of uncertainty by boiling it, filtering it and putting chlorine or bleach in it^20^. Moreover, other practices include frequent washing of hands with water and soap from a decent, reliable source before, during, and after food handling as well as after the usage of the toilet^20^,^21^. Both the WHO and the Centers for Disease Control and Prevention (CDC) commend that individuals living in high-risk and outbreak areas or individuals who will travel to endemic countries take the oral cholera vaccines^22^. These vaccines, which consist of the killed strains of *V. cholerae*, provide for up to 2 years, notable immunity in both children and adults^22^. Furthermore, USA and Europe alone have approved a live attenuated oral cholera vaccine, that showed to instigate by one oral dose, a more rapid immune response; however, additional exploration is needed to study its advancement in the community^22^. In the long-term incorporation of strategies for responsible finance and resource procurement, advancement of diagnostics, and worldwide cooperation are crucial to promoting the sustainable improvement of healthcare services in Africa^23^.

## Conclusion

The re-emergence of cholera outbreaks in African countries like Kenya continues, resulting in high morbidity and mortality. In this study, the risk factors contributing to the current outbreak were assessed stating the importance of water sanitation, food hygiene and the adoption of multisector approaches for disease prevention and control. The improvement of healthcare facilities is needed to ensure prompt diagnosis, rapid response and treatment in case of future epidemics. Vaccination should be advocated for all high-risk groups in cholera-endemic areas and public health interventions should be strengthened. Epidemiological surveillance systems and socioeconomic support should be extended to intensify efforts on active case monitoring and statistical data collection for scientific analysis. This would broaden our knowledge through further research and help in the implementation of control strategies for the effective use of scarce resources in the management of this infectious disease.

## Ethical approval

Not applicable.

## Sources of funding

None.

## Authors’ contribution

O.U.: conceptualization, project administration, writing – review and designing. All authors were involved in manuscript writing, data collection, and assembly; final approval of manuscript.

## Conflicts of interest disclosure

The authors declare that they have no financial conflict of interest with regard to the content of this report.

## Research registration unique identifying number (UIN)

None.

## Guarantor

Abubakar Nazir.
